# Atrial Fibrillation and Perioperative Inflammation (FIBRILLAMMED Study): A Retrospective Analysis of the Predictive Role of Preoperative Albumin-Adjusted Platelet-Leukocytic Indices in OPCABG

**DOI:** 10.4274/TJAR.2023.22995

**Published:** 2023-08-18

**Authors:** Rohan Magoon, Iti Shri, Ramesh C. Kashav, Souvik Dey, Jasvinder K. Kohli, Vijay Grover, Vijay Gupta

**Affiliations:** 1 Department of Anaesthesia, Atal Bihari Vajpayee Institute of Medical Sciences & Dr. Ram Manohar Lohia Hospital, New Delhi, India; 2 Department of Cardiac Anaesthesia, Atal Bihari Vajpayee Institute of Medical Sciences & Dr. Ram Manohar Lohia Hospital, New Delhi, India; 3 Department of Cardiothoracic Vascular Surgery, Atal Bihari Vajpayee Institute of Medical Sciences & Dr. Ram Manohar Lohia Hospital, New Delhi, India

**Keywords:** Albumin, coronary artery bypass grafting, neutrophil-to-lymphocyte ratio, new-onset atrial fibrillation, perioperative inflammation, platelet-to-lymphocyte ratio, systemic immune-inflammation index

## Abstract

**Objective:**

New-onset atrial fibrillation (NOAF), an important postoperative complication, has pertinent inflammatory links. Motivated by the encouraging literature on the prognostic role of hypoalbuminemia, leukocytic indices [LIs: neutrophil-to-lymphocyte ratio (NLR), monocyte-to-lymphocyte ratio (MLR)], systemic inflammation response index (SIRI=NLR×monocyte) and platelet-leukocytic indices [PLIs: platelet-to-lymphocyte ratio (PLR)], systemic immune inflammation index (SII=NLR×platelet), aggregate index of systemic inflammation (AISI=NLR×platelet×monocyte), we sought to investigate the NOAF-predictive value of preoperative albumin-adjusted indices (aa-LIs and aa-PLIs) in an off-pump coronary artery bypass grafting (OPCABG) setting.

**Methods:**

Of 899 patients, 151 patients (16.79%) developed the primary outcome i.e. NOAF that was analyzed further retrospectively for its predictors instead of the highlighted text perioperative data of 899 patients undergoing elective OPCABG, were retrospectively analyzed. The study participants were categorized into non-NOAF and NOAF groups (defined as new-onset atrial arrhythmia with irregular RR interval with indistinct *P* wave in the first week postoperatively).

**Results:**

One hundred and fifty-one patients (16.79%) developed NOAF. On univariate analysis: age, smoker status, The European System for Cardiac Operative Risk Evaluation (EuroSCORE) II, systemic hypertension, diabetes mellitus, prior congestive heart failure (CHF), and a higher preoperative NLR, PLR, SII, and albumin were significant predictors of NOAF. While age, CHF, and EuroSCORE II retained predictive significance in multivariate analysis, LI-PLIs and albumin did not emerge as independent NOAF predictors. Notably, aa-NLR, aa-PLR, and aa-SII independently predicted NOAF on the computation of model-estimates in the regression analysis (Odds ratio; 95% confidence interval: 31.05;15.75-70.61, 1.04;1.02-1.05, 1.12;1.10-1.14, respectively, *P* < 0.001). aa-NLR ≥1.32, aa-PLR ≥52.64, and aa-SII ≥344.38 predicted NOAF with the respective AUC;sensitivity;specificity of 0.66;63.6%;73.3%, 0.63;66.2%;59.0%, and 0.65;58.3%;78.2%. Preoperative aa-NLR, aa-PLR and aa-SII also positively correlated with CHA_2_DS_2_-VASc score (R=0.40, 0.45 and 0.42; *P* < 0.001).

**Conclusion:**

The independent NOAF predictive value of aa-NLR, aa-PLR, and aa-SII reiterates the inflammatory relationship of the arrhythmic complication following OPCABG.

**Main Points**

• New-onset atrial fibrillation (NOAF), a common complication (15-36%) following coronary artery bypass grafting (CABG) has relevant inflammatory etiology.

• Literature is accruing that emphasize the NOAF predictive and prognostic value of inflammatory hematological parameters viz. leukocytic indices (LIs), and platelet-leukocytic indices (PLIs) following cardiac surgery.

• There also exist some studies suggestive of possible role of hypoalbuminemia in predicting AF.

• Therefore we retrospectively evaluated our large set of off-pump CABG data to evaluate the NOAF predictive value of albumin adjusted LIs and PLIs.

## Introduction

New-onset atrial fibrillation (NOAF), frequently compounds the postoperative course following coronary artery bypass grafting (CABG) with an overall reported incidence of 15-36%, entailing a substantial postoperative morbidity-mortality.^1,2^ Hence, NOAF risk-stratification constitutes an area of active research interest, particularly in order to efficiently prioritize preventive and/or therapeutic interventions.

In this context, perioperative inflammation is being increasingly scrutinized for its’ role in attributing a higher risk of NOAF.^2,3^ This is largely motivated by the pathophysiological proposition of an enhanced atrial conduction “anisotropy” owing to a pro-inflammatory milieu and the concomitant description of novel promising parsimonious inflammatory biomarkers.^2^ With regards to inflammatory prognostication, encouraging literature is accumulating on the AF-predictive role of leukocytic ratios such as neutrophil-to-lymphocyte ratio (NLR) and platelet-leukocytic ratios and indices [platelet-to-lymphocyte ratio (PLR)] and systemic immune inflammation index (NLR×platelet), from diverse operative and non-operative predilected settings.^4,5,6,7,8,9^

Hypoalbuminemia too has considerable significance from the inflammatory standpoint and its’ association with poor cardiac surgical outcomes (including NOAF).^10,11^ Recent literature points towards co-existence of hypoalbuminemia with a pro-inflammatory leukocytic alteration pattern conferring a likely incremental prognostic value to the composite evaluation of the parameters.^10,12,13^ Therefore, we sought to retrospectively evaluate the NOAF predictive value of albumin-adjusted leukocytic indices [aa-LIs, including aa-NLR, monocyte-to-lymphocyte ratio (aa-MLR), and aa-systemic inflammation response index (aa-SIRI=NLR×monocyte/albumin)] and platelet-leukocytic indices [aa-PLIs, including aa-PLR, aggregate index of systemic inflammation (aa-AISI=NLR×platelet×monocyte/albumin)] and albumin adjusted systemic immune inflammation index (aa-SII=NLR×platelet/albumin) in our study aiming for a simultaneous account of AF and perioperative inflammation (FIBRILLAMMED study) in patients undergoing off-pump CABG (OPCABG). The secondary objectives of the study were to derive the NOAF predictive cut-offs of aa-LIs and aa-PLIs and evaluate the correlation of preoperative hematological inflammatory indices with the CHA_2_DS_2_-VASc score^14^ (a scoring system widely described to be associated with an elevated NOAF-risk).

## Methods

After obtaining the ethical approval by Institutional Ethics Committee of Atal Bihari Vajpayee Institute of Medical Sciences & Dr. Ram Manohar Lohia Hospital [554(90/2021)/IEC/ABVIMS/RMLH/735], the present retrospective case-control study was conducted at our tertiary care referral center, on patients scheduled for elective OPCABG surgery between January 2017 and October 2021. Patients with pre-existing arrhythmia or amiodarone therapy, pre-existing neurological deficit, thyroid disorder, anemia with hemoglobin (Hb) <10.0 g dL^-1^, unavailability of hematological investigations within 72 hours of surgery, hepatic dysfunction [serum glutamic-oxaloacetic transaminase (SGOT), and serum glutamic pyruvic transaminase (SGPT) greater than twice the baseline], end-stage renal disease (glomerular filtration rate <30 mL min^-1^ or hemodialysis), undergoing either concomitant valve surgery and emergency CABG were excluded at the beginning of the study. Apart from these any condition affecting leukocyte counts and albumin levels were also excluded eg. active infection, patient on steroids, lymphoproliferative disorders, or systemic hypoalbuminemic disorder.

Preoperative demographical characteristics evaluated were age, sex, history of smoking, hypertension (HTN), diabetes mellitus (DM), chronic obstructive pulmonary disease (COPD), peripheral vascular disease (PVD), history of prior congestive heart failure (CHF), history of previous myocardial infarction (MI), hyperlipidemia. The European System for Cardiac Operative Risk Evaluation (EuroSCORE) II was evaluated for each patient from the calculator available on official website www.euroscore.org. A detailed preoperative drug history was also recorded. Preoperative laboratory investigations compared between the groups were Hb, total leukocyte count, differential leukocyte count (DLC), platelet count, blood urea, serum creatinine, albumin, SGOT and SGPT levels, triglycerides, and on echocardiogram, left-ventricular ejection fraction and regional wall motion abnormality were noted. From the DLC, NLR, PLR, MLR, SII, SIRI and the AISI values were derived.^15^ The albumin-adjusted LIs and PLIs were computed by dividing the corresponding values by serum albumin levels, eg: aa-NLR=NLR/albumin. Intraoperatively duration of surgery, the total number of grafts, vessel grafted, and number of blood and blood product units transfused were noted.

Postoperatively, the parameters recorded were duration of mechanical ventilation (DOMV), length of intensive care unit (LOS-ICU) stay, length of hospital stay (LOS-H), postoperative mean lactate levels, mean vasoactive inotropic score (VIS), incidence of major adverse cardiac events (MACE), cerebrovascular accident (CVA), acute kidney injury (AKI), intra-aortic balloon pump (IABP) insertion, and in-hospital mortality.

VIS was calculated as dopamine (µg kg^-1^ min^-1^)+dobutamine (µg kg^-1^ min^-1^)+milrinone (µg kg^-1^ min^-1^)*×*10+ epinephrine (µg kg^-1^ min^-1^)*×*100+norepinephrine (µg kg^-1^ min^-1^)*×*100+ vasopressin (µg kg^-1^ min^-1^)*×*10000.^15^

MACE was characterized by any of the following: ST-segment elevation Myocardial Ischemia, low cardiac-output syndrome (LCOS, cardiac index <1.5 L min^-1^ m^2^), and cardiac-arrest.^15^ Perioperative CVA was defined as any new temporary or permanent, focal or global neurologic deficit in accordance with the standardized Valve Academic Research Consortium-2 guidelines for cerebrovascular events after Trans Catheter Aortic Valve Implantation.^16^ New-onset renal failure was defined according to Kidney Disease: Improving Global Outcomes Foundation as an increase in serum creatinine by ≥0.3 mg dL^-1^ (≥26.5 µmol L^-1^) within 48 h; or an increase in serum creatinine to ≥1.5 times the baseline value, which is known or presumed to have occurred within the prior 7 days; or urine volume < 0.5 mL kg^-1^ h^-1^ for 6 h.^17^

CHA_2_DS_2_-VASc score consists of CHF, HTN, age (between 64-75, and if above 75 assigned a score of 2), diabetes, sex, history of stroke (assigned a score of 2), and vascular disease. Higher the CHA_2_DS_2_-VASc score, higher the stress on the atria and hence an increased incidence of NOAF and subsequent stroke.^14^

The anaesthetic induction and maintenance were as per institutional protocol. After standard premedication, arterial line was inserted and the patient was induced with 3-5 µg kg^-1^ fentanyl, 0.2 mg kg^-1^ etomidate, and 1.2 mg kg^-1^ rocuronium, titrated to hemodynamics. Patient was put on volume control mechanical ventilation at a ratio of inspired oxygen and air at 1:1, tidal volume 6-8 mL kg^-1^ to maintain end-tidal carbon dioxide at 34-35 mmHg. Central venous line and pulmonary artery catheter were placed. Anaesthesia was maintained with fentanyl, rocuronium, and isoflurane. Arterial blood gas analysis was performed at regular intervals to monitor arterial oxygen concentration, electrolyte balance, mixed venous oxygen saturation (ScVO_2_), Hb and hematocrit, and blood sugar levels. Temperature was monitored and maintained at 35-36°C. Blood sugar values were maintained between 140-180 mg dL^-1^ by using insulin infusion if required. Blood and blood products were transfused if Hb below 10 g dL^-1^ or hematocrit <28-30% and based on Sonoclot ACT machine results respectively.

After midline sternotomy, the left internal mammary artery (LIMA) was harvested. Simultaneously saphenous venous graft too was harvested. Heparin 200 IU kg was administered after LIMA harvest to achieve an activated clotting time of >300 sec. The Octopus Evolution Tissue Stabilizer (Medtronic, Inc, Minneapolis, MN) was used to stabilize the target coronary artery and after placing intracoronary shunts as to prevent distal ischemia and maintain graft patency, distal anastomosis was performed with 7-0 prolene suture, and for proximal anastomosis 5-0 prolene sutures were used.

At the end of anastomosis, heparin was reversed with protamine in a ratio of 1 mg/100 IU of heparin and the patient shifted to ICU for elective mechanical ventilation, and extubated once the extubation criteria were satisfied. Strict hemodynamic monitoring was followed in the ICU using invasive pressure monitoring and rhythm monitoring. In addition, a 12-lead electrocardiogram was performed daily, and on the detection of arrhythmias. Occurrence of NOAF was defined as a new onset atrial arrhythmia with irregular RR intervals without discernible *P* wave on 12 lead ECG or as recorded in the case files, in the first 7 days postoperatively.^18^

### Statistical Analysis

For descriptive statistics, after dividing the patients into NOAF and non-NOAF groups, continuous variables are expressed as mean, median, and standard deviation and compared across the groups using Mann-Whitney U test (on applying Shapiro-Wilk’s test, the data was appropriate for non-parametric tests and hence Mann-Whitney U test was used employing respective median values), while categorical variables are expressed as number and percentage of patients and compared across the groups using Pearson’s chi-square test for independence. Odds ratio (OR), 95% confidence interval (CI) and significance level is provided for variables significant in the univariate logistic regression analysis. Multivariate analysis was done using binary logistic regression. Furthermore, for the analysis of albumin-adjusted parameters three multivariate binary logistic regression analysis models were designed for variables discriminating two groups. The threshold value effect of albumin, each hematological parameter, and albumin-adjusted ratios on NOAF was observed by receiver operating characteristic (ROC) curve, and calculated relative predictive powers as measured by area under the curve (AUC). The sensitivity, specificity, and predictive values were reported using these generated cut-offs. Association between continuous variables is captured by Spearman’s rank correlation coefficient. The statistical software SPSS version 22 has been used for the analysis. An alpha level of 5% i.e. any *P* value less than 0.05 is considered significant.

## Results

**Figure 1:**
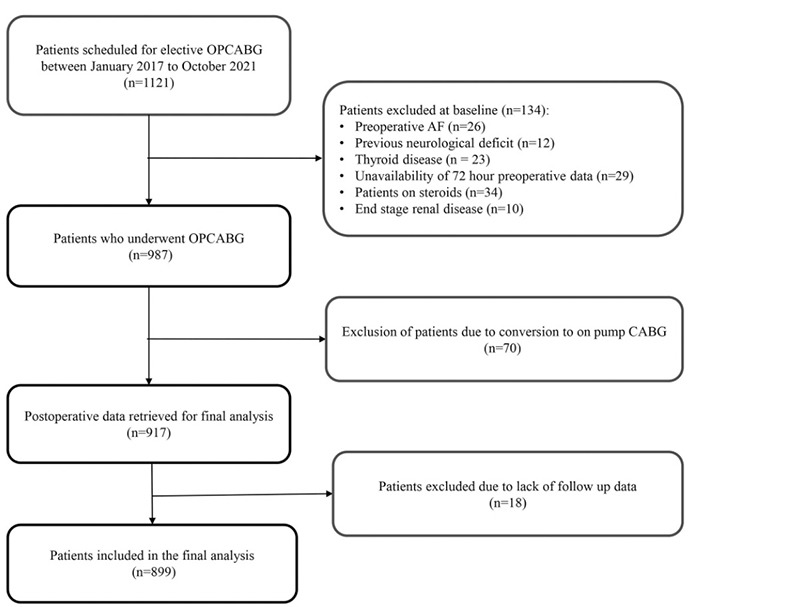
Flowchart depicting patient enrolment. OPCABG, off-pump coronary artery bypass grafting; AF, atrial fibrillation.

Out of total 1121 patients initially inducted, after accounting for the exclusion of 134 patients at the baseline, 70 patients due to intraoperative conversion to on-pump, and concomitant unavailability of adequate follow up data in 18 patients; 899 patients were eventually included in the study. The patient enrolment is illustrated in Figure 1. Of these, 151 (16.79%) developed NOAF. The perioperative variables including patient demographics, comorbidities, preoperative investigations, and intraoperative parameters were compared between the NOAF and non-NOAF groups, as depicted in Table 1.

Subsequent to univariate regression analysis, factors predicting NOAF after OPCABG were: advanced age, higher EuroSCORE II, smoker status, comorbidities viz systemic HTN, DM, prior CHF, and a higher NLR, PLR, SII and serum albumin level as outlined in Table 2. A multivariate regression analysis was further computed using the aforementioned parameters to ascertain variables independently affecting occurrence of NOAF. It was found that age (OR: 1.12; 95% CI: 1.07-1.17; *P* < 0.001), EuroSCORE II (OR: 1.95; 95% CI: 1.53-2.48; *P* < 0.001), and history of prior CHF (OR: 2.43; 95% CI: 1.43-4.11; *P*=0.001) were independent predictors of NOAF (Table 2). However, albumin and the leukocytic parameters (serum albumin and LI-PLIs) were non-significant following multivariate regression analysis, as illustrated in Table 2. Hence, model estimates were calculated for aa-LIs and aa-PLIs, after adjusting the respective NLR, PLR, and SII values for the corresponding serum albumin levels as outlined in Table 3. In model aa-NLR, it is our variable of interest and other confounding variables found significant on univariate analysis were adjusted with aa-NLR to assess if it can independently predict the occurrence of NOAF. Similarly in models B and C, confounding variables were adjusted with aa-PLR and aa-SII respectively. After computation of model estimates using aa-LIs and aa-PLIs and other parameters predicting NOAF on univariate analysis, aa-NLR, aa-PLR, and aa-SII independently predicted the occurrence of NOAF (OR; 95% CI: 31.05; 15.75-70.61, 1.04; 1.02-1.05, 1.12; 1.10-1.14, respectively; *P* < 0.001).

**Figure 2:**
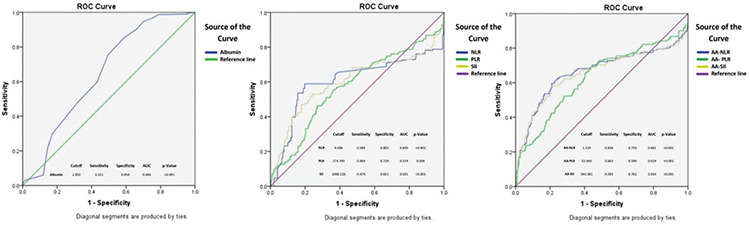
The receiver operating characteristic curve depicting cut off values with sensitivity, specificity and area under the curve of albumin, leukocytic and platelet leukocytic indices, albumin adjusted leukocytic indices for predicting NOAF. ROC, receiver operating characteristic; AUC, area under the curve; NOAF, new onset atrial fibrillation; aa-NLR, albumin adjusted neutrophil-lymphocyte ratio; aa-PLR, albumin adjusted platelet-lymphocyte ratio; aa-SII, albumin adjusted systemic immune inflammation index.

Subsequently, ROC analysis of serum albumin, LI-PLIs revealed the cut-off values for the development of NOAF following OPCABG as illustrated in Figure 2. The derived respective cut-off values were: serum albumin ≤2.85 (sensitivity: 41.1%, specificity: 85.4%, AUC: 0.646), NLR ≥4.01 (AUC: 0.605; sensitivity: 58.9%, specificity: 80.2%), PLR ≥174.74 (AUC: 0.574; sensitivity: 46.4%, specificity: 72.9%), SII ≥1066.23 (AUC: 0.601; sensitivity: 47.0%, specificity: 82.1%). The cut-off values of aa-LI and aa-PLIs were: aa-NLR ≥1.32 (AUC: 0.661; sensitivity: 63.6%, specificity: 73.3%), aa-PLR ≥52.64 (AUC: 0.629; sensitivity: 66.2%, specificity: 59.0%) and aa-SII ≥344.38 (AUC: 0.654; sensitivity: 58.3%, specificity: 78.2%). Of note is that the AUC values for the aa-ratio indices were higher as compared to the lone ratio-indices. Secondarily, it was also found that the patients in the NOAF group had a significantly escalated incidence of poor outcomes as depicted in Table 4. Patients in the NOAF group had a significant increase in the incidence of MACE, CVA, AKI, LCOS, and the requirement of IABP. Similarly, NOAF was also found to significantly increase DOMV, LOS-ICU, and LOS-H with a higher postoperative mean lactate level, and mean-VIS score value. The in-hospital mortality, however was similar in both groups (1.99% versus 1.07%, *P*=0.350).

**Figure 3:**
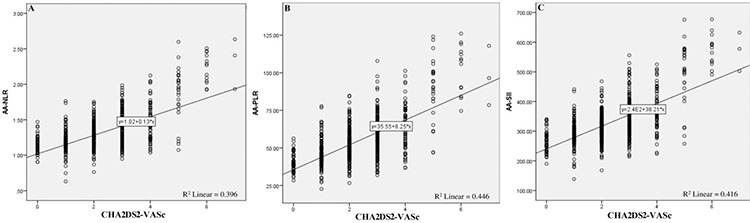
Spearman’s rank correlation coefficient analysis of aa-NLR, aa-PLR, and aa-SII with CHA_2_DS_2_-VASc score. aa-NLR, albumin adjusted neutrophil-lymphocyte ratio; aa-PLR, albumin adjusted platelet-lymphocyte ratio; aa-SII, albumin adjusted systemic immune inflammation index.

On applying Spearman’s correlation analysis between CHA_2_DS_2_VASc, and aa-LIs and aa-PLIs, a significant linear correlation (aa-NLR: R=0.40, aa-PLR: R=0.45, aa-SII: R=0.42; *P* < 0.001) was revealed between the two parameters as depicted in Figure 3.

## Discussion

The index elucidation of an independent NOAF-predictive significance of the aa-LIs and aa-PLIs in an OPCABG setting (ahead of the albumin and LI-PLIs alone), is congruent with the recent studies from non-operative settings suggesting an augmented inflammatory prognostic potential of a combined account of the leukocytic and albumin levels.^13,19^ The 16.79% incidence of NOAF in our study relates closely to the existing literature on the research subject.^1^ As far as postoperative NOAF predisposition is concerned, the risk-factors emerging from our retrospective analysis are also largely comparable to the NOAF literature in cardiac surgical arena.^14,20^

Inflammatory conditions such as pericarditis, postoperative period, and histological findings of inflammatory infiltrates in cases of lone AF suggest inflammation is intimately associated with the occurrence of AF, the major research element in the index study.^21^ The postoperative inflammatory atrium is “anisotropic” i.e. variegated with cells having different refractory periods and conduction velocities, enhancing its susceptibility to aberrant electrical activity.^2^ A 2014 systematic review by Jacob et al.^3^ highlighted the importance of white blood cell elevations in predicting NOAF after cardiac surgery. Furthermore, the 2020 meta-analysis by Liu et al.^4^ distinctively addressed the research subject of NLR predictive value of AF in cardiac surgery by a pooled analysis of 12 studies and 9,262 patients. They outlined preoperative NLR as a significant predictor of NOAF (OR: 1.42, 95% CI: 1.16-1.72) and concluded that the former emerges as a promising NOAF prognostic marker but simultaneously flagged the residual potential sources of heterogeneity.^4^ Our study also delineated preoperative NLR as a NOAF predictor in univariate but not in multivariate analysis. The preoperative NLR cut-off of 4.01 derived in our study, also corroborates with the threshold NLR values described in the studies included in the Liu et al.^4^ meta-analysis. Interestingly, the Liu et al.^4^ meta-analysis additionally demonstrated that an elevated postoperative NLR fails to predict NOAF. Meanwhile, OPCABG is considered to be associated with lower incidence of NOAF in view of the absence of side effects of extracorporeal circulation and inadequate myocardial protection as well as due to advances in the OPCABG techniques.^22^ The incidence of AF in our study is 16.79%, commensurate with that in the literature and less than on-pump CABG which is 21.7%.^22^

Ahead of NLR, PLR and SII have received research attention in the purview of NOAF prediction following cardiac surgery. A preoperative NOAF-predictive PLR cut-off of 174.74 emanating from our study exceeds the 119.3 cut-off of PLR outlined by Gungor et al.^5^. Nonetheless, there is a dearth of literature on PLR cut-offs for post-cardiac surgery NOAF prediction in order to draw holistic comparisons. As far as preoperative SII is concerned, our cut-off value of 1066.22 mm^3^ adds to the recent studies by Selcuk et al.^7^ and, Ata and Abanoz^23^ highlighting NOAF-predictive SII cut-offs of 807.8 mm^3^ and 986 mm^3^, in their respective operative settings of isolated CABG.

Specific to the post-CABG NOAF links of hypoalbuminemia, Akgül et al.^11^ outlined significantly lower albumin levels of 2.87 ± 0.34 g dL^-1^ in NOAF-patients versus 3.77 ± 0.47 g dL^-1^ in non-NOAF patients. The corresponding NOAF-predictive cut-off was 3.05 g dL^-1^ in the Akgül et al.^11^ study whereas 2.85 g dL^-1^ emerged as the NOAF-predictive albumin cut-off in our OPCABG cohort. More importantly, the higher OR of C-reactive protein (CRP) albumin ratio (OR: 1.85; 95% CI: 1.60-2.14; *P* < 0.001) in comparison to the serum CRP and albumin levels alone (OR: 1.16; CI: 1.11-1.20; *P* < 0.001; OR: 0.44; CI: 0.26-0.86; *P* < 0.001, respectively) outlined by Karabacak et al.^24^ in 830 CABG patients, epitomizes the basic concept of our study propounding the need for a comprehensive inflammatory assessment.

Appropriate to the context of the prognostic value-addition owing to a combined account for hypoalbuminemia and inflammatory leukocytic alterations, Zhang et al.^13^ recently highlighted the association of systemic inflammation score (SIS, a score computed by relative weight analysis of albumin and leukocytic ratios) with AF in 376 pairs of cases and controls using a propensity score matching system. However, our study evaluated a homogeneous surgical cohort (CABG) and adjusted the individual leukocytic ratios and the indices to the albumin levels motivated by the recent Yoon et al.^12^ elucidation of the co-existence of lower albumin levels with higher SII, in cardiac surgical patients.

The perioperative inflammatory response plays a pivotal role in the genesis of NOAF.^3,4^ Alongside the comprehension of an inflammatory alliance of neutrophilia and lymphocytopenia, albumin also has vital physiological functions including the maintenance of vascular endothelial integrity, anti-oxidative and anti-inflammatory properties.^10,11,13,24,25^ From a NOAF-pathophysiological perspective, perioperative inflammation renders the myocardium as a tissue mosaic of varying refractory periods and/or conduction velocities which enhances the susceptibility to aberrant electrical-activity, and conduction re-entry - the so called “anisotropic” atrium.^2^

### Study Limitations

To the best of our knowledge, the index study classifies as a seminal research endeavour focusing on the NOAF-predictive value of aa-LIs and aa-PLIs in OPCABG. Needless to say, the large homogeneous surgical cohort is the major strength of the study. Moreover, the additional evaluation of the correlation of the preoperative aa-LIs and aa-PLIs with well-established patient-centric NOAF-risk scoring systems like the CHA_2_DS_2_-VASc score, adds further credibility to the research findings. It is worthwhile to point out that the CHA_2_DS_2_-VASc score employs certain patient-risk factors such as age (which are continuous parameters, speaking strictly statistically) as categorical parameters for the scoring purposes (eg: age >75 years: scored 2 and age 65-74 years scored as 1).^14^ Nonetheless, we studied such risk-parameters in their original continuous statistical connotations.

The study had its’ own limitations given the retrospective design being peculiarly susceptible to residual confounding.^26^ Although, as mentioned above, the Liu et al.^4^ meta-analysis failed to attribute NOAF-predictive value to the postoperative NLR, the inclusion of postoperative LIs and PLIs in our study could have rendered the analysis more comprehensive. With that said, the isolated evaluation of an off-pump patient subset in our study potentially prevents the hematological and biochemical perturbations associated with the conduct of extracorporeal circulation. The lack of echocardiographic data on the concomitant atrial dimensions of the study participants, is also an important limitation considering NOAF was being evaluated as the primary outcome.^27^ Lastly, inclusion of other markers like CRP could have further strengthened the inflammatory relationship proposed in the present research endeavour.^24^

## Conclusion

The independent NOAF predictive value of aa-LIs and aa-PLIs reiterates the inflammatory relationship of the arrhythmic complication following cardiac surgery. While awaiting future prospective literature in this area of clinical importance, it only becomes imperative to reflect upon the need of an inflammatory account in the comprehensive risk-stratification and subsequent positive risk-modulation, aligned with the ultimate goal of improving postoperative outcomes in cardiac surgical subset.

## Figures and Tables

**Table 1:**
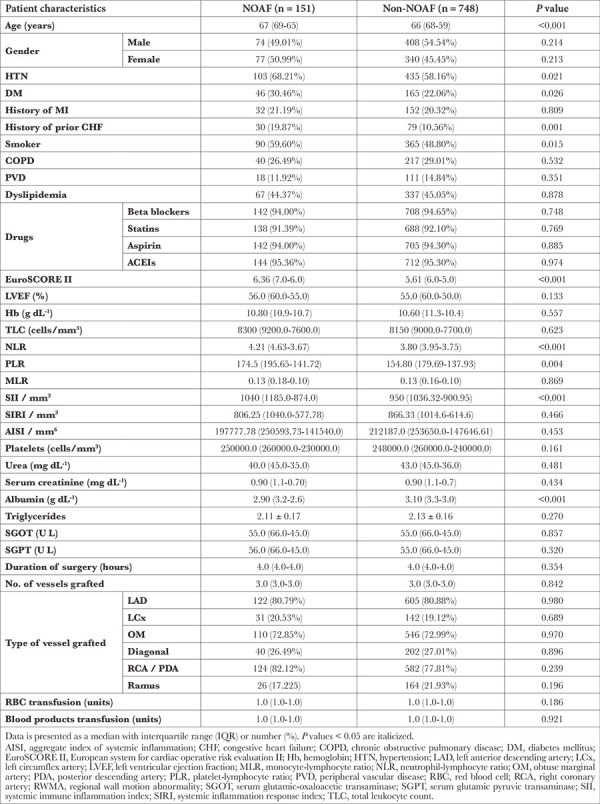
Comparison of Baseline Demographic and Laboratory Parameters, and Intraoperative Parameters Between NOAF and Non-NOAF Groups

**Table 2:**
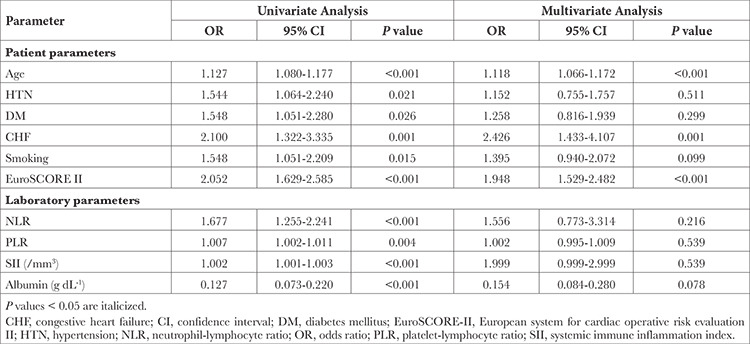
Univariate and Binary Logistic Regression Analysis of Parameters Predicting NOAF

**Table 3:**
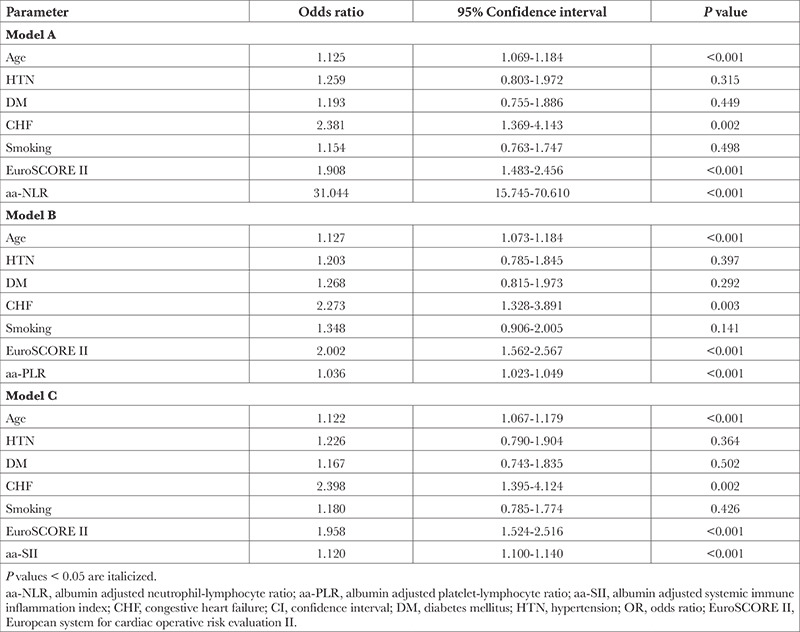
Models for Individual NOAF Predictive Ability of Albumin Adjusted Leukocytic Indices

**Table 4:**
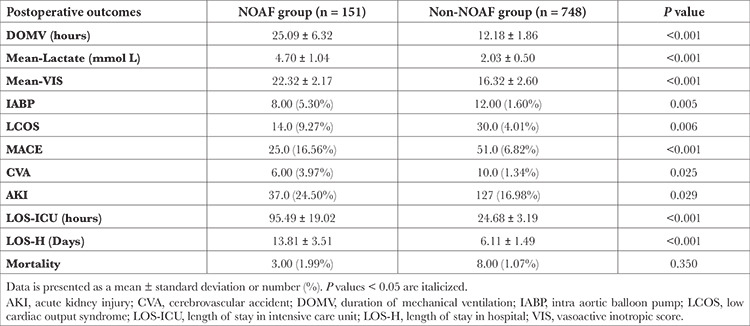
Comparison of Postoperative Outcomes in the NOAF and Non-NOAF Groups

## References

[1] Kerwin M, Saado J, Pan J, et al (2020). New-onset atrial fibrillation and outcomes following isolated coronary artery bypass surgery: A systematic review and meta-analysis. Clin Cardiol..

[2] Mostafa A, El-Haddad MA, Shenoy M, Tuliani T (2012). Atrial fibrillation post cardiac bypass surgery. Avicenna J Med..

[3] Jacob KA, Nathoe HM, Dieleman JM, van Osch D, Kluin J, van Dijk D (2014). Inflammation in new-onset atrial fibrillation after cardiac surgery: a systematic review. Eur J Clin Invest..

[4] Liu Z, Nguyen Khuong J, Borg Caruana C, et al (2020). The Prognostic Value of Elevated Perioperative Neutrophil-Lymphocyte Ratio in Predicting Postoperative Atrial Fibrillation After Cardiac Surgery: A Systematic Review and Meta-Analysis. Heart Lung Circ..

[5] Gungor H, Babu AS, Zencir C, et al (2017). Association of Preoperative Platelet-to-Lymphocyte Ratio with Atrial Fibrillation after Coronary Artery Bypass Graft Surgery. Med Princ Pract..

[6] Şaşkın H, Düzyol Ç, Özcan KS, Aksoy R, Idiz M (2015). Preoperative Platelet to Lymphocyte Ratio Is Associated with Early Morbidity and Mortality after Coronary Artery Bypass Grafting. Heart Surg Forum..

[7] Selcuk M, Cinar T, Saylik F, Dogan S, Selcuk I, Orhan AL (2021). Predictive Value of Systemic Immune Inflammation Index for Postoperative Atrial Fibrillation in Patients Undergoing Isolated Coronary Artery Bypass Grafting. Medeni Med J..

[8] Bağcı A, Aksoy F (2021). Systemic immune-inflammation index predicts new-onset atrial fibrillation after ST elevation myocardial infarction. Biomark Med..

[9] Luo Y, Zhang J, Liu T, et al (2022). The systemic-immune-inflammation index predicts the recurrence of atrial fibrillation after cryomaze concomitant with mitral valve surgery. BMC Cardiovasc Disord..

[10] Magoon R, Shri I, Jose J (2022). The malnutritional facet of inflammatory prognostication in acute aortic dissection. J Card Surg..

[11] Akgul E, Parlar AI, Erkul GSA, Erkul S, Cekirdekci A (2020). Investigation of the Effect of Preoperative Hypoalbuminemia, Blood Urea Nitrogen and Creatinine Levels on Postoperative Atrial Fibrillation on Off-Pump Coronary Bypass Surgery Patients. Heart Surg Forum..

[12] Yoon J, Jung J, Ahn Y, Oh J (2021). Systemic Immune-Inflammation Index Predicted Short-Term Outcomes in Patients Undergoing Isolated Tricuspid Valve Surgery. J Clin Med..

[13] Zhang H, Li J, Chen X, et al (2018). Association of Systemic Inflammation Score With Atrial Fibrillation: A Case-Control Study With Propensity Score Matching. Heart Lung Circ..

[14] Chua SK, Shyu KG, Lu MJ, et al (2013). Clinical utility of CHADS2 and CHA2DS2-VASc scoring systems for predicting postoperative atrial fibrillation after cardiac surgery. J Thorac Cardiovasc Surg..

[15] Dey S, Kashav R, Kohli JK, et al (2021). Systemic Immune-Inflammation Index Predicts Poor Outcome After Elective Off-Pump CABG: A Retrospective, Single-Center Study. J Cardiothorac Vasc Anesth..

[16] Devgun JK, Gul S, Mohananey D, et al (2018). Cerebrovascular Events After Cardiovascular Procedures: Risk Factors, Recognition, and Prevention Strategies. J Am Coll Cardiol..

[17] Park JT (2017). Postoperative acute kidney injury. Korean J Anesthesiol..

[18] Gibson PH, Cuthbertson BH, Croal BL, et al (2010). Usefulness of neutrophil/lymphocyte ratio as predictor of new-onset atrial fibrillation after coronary artery bypass grafting. Am J Cardiol..

[19] Kao HK, Löfstrand J, Loh CY, et al (2018). Nomogram based on albumin and neutrophil-to-lymphocyte ratio for predicting the prognosis of patients with oral cavity squamous cell carcinoma. Sci Rep..

[20] Burgos LM, Ramírez AG, Seoane L, et al (2021). New combined risk score to predict atrial fibrillation after cardiac surgery: COM-AF. Ann Card Anaesth..

[21] Guo Y, Lip GY, Apostolakis S (2012). Inflammation in atrial fibrillation. J Am Coll Cardiol..

[22] Wu CY, Wang SH, Shang YQ, Xia JH (2017). Incidence of atrial fibrillation after off-pump versus on-pump coronary artery bypass grafting: A meta-analysis of randomized clinical trials and propensity score matching trials. J Huazhong Univ Sci Technolog Med Sci..

[23] Ata Y, Abanoz M (2021). Predictive Roles of Right Coronary Artery Disease Severity and Systemic Immune Inflammation Index in Predicting Atrial Fibrillation After Coronary Bypass Operations in Patients with Right Coronary Artery Disease. Heart Surg Forum..

[24] Karabacak K, Kubat E, Akyol FB, et al (2020). The C-reactive protein/albumin ratio as a new predictor for postoperative atrial fibrillation after coronary artery bypass graft surgery. J Card Surg..

[25] Magoon R, Jose J (2022). Letter to the Editor: “Peripheral blood neutrophil-to-lymphocyte ratio is associated with mortality across the spectrum of cardiogenic shock severity”. J Crit Care..

[26] Magoon R, Jose J (2020). Safeguarding anaesthesia research from spin. Br J Anaesth..

[27] Cho MS, Park HS, Cha MJ, et al (2021). Clinical impact of left atrial enlargement in Korean patients with atrial fibrillation. Sci Rep..

